# Post-error behavioral adjustments under reactive control among older adults

**DOI:** 10.3389/fpsyg.2022.1001866

**Published:** 2022-11-01

**Authors:** Noriaki Tsuchida, Ayaka Kasuga, Miki Kawakami

**Affiliations:** ^1^College of Comprehensive Psychology, Ritsumeikan University, Osaka, Japan; ^2^Graduate School of Human Sciences, Osaka University, Osaka, Japan; ^3^Institute of Human Sciences, Ritsumeikan University, Osaka, Japan

**Keywords:** aging, error, post-error behavior, older adults, reactive control

## Abstract

This study analyzed the effects of aging on post-error behavioral adjustments from the perspective of cognitive control. A modified error awareness task was administered to young (*n* = 50) and older (*n* = 50) adults. In this task, two buttons were placed on the left and right sides in front of the participants, who were instructed to use the right button to perform a go/no-go task, and were notified if they made an error. There were three experimental conditions (A, B, and C): participants had to push the right button once in Condition A and twice in Condition B and C when a go-stimulus was presented. Conversely, participants were asked to withhold their response when a no-go stimulus was presented. Response inhibition differed depending on the experimental condition. The participants were asked to push the left button as quickly as possible when an error occurred. The results showed relatively longer reaction times to sudden errors among older adults compared with young adults. Furthermore, the difference in the error responses (i.e., accidentally pushing the right button once or twice when a no-go stimulus was presented) strongly influenced older adults’ response time after an error. These results suggest that the shift from proactive to reactive control may significantly influence post-error behavioral adjustments in older adults.

## Introduction

Accidents caused by errors committed by older adults have recently received academic attention (Tefft, 2017). As a specific example, On April 19 of 2019, an elderly adult had a car accident in Ikebukuro, Tokyo (The Asahi Shimbun, 2019). Reviewing the process of the accident made it apparent that the older adult continued to cause many accidents simultaneously because he could not respond after the first accident (i.e., they may be unable to step on the brake pedal quickly), which resulted in a severe incident. Furthermore, many precedents have shown that cognitive control functions generally decline with age (e.g., inhibition: Hasher and Zacks, 1988; Dempster, 1992; cognitive control modes: Braver, 2012; van Gerven et al., 2016). It is assumed that there were some kind of cognitive control problems behind the accidents. Therefore, focusing on post-error adjustments from the viewpoint of cognitive control is essential when investigating errors committed by older adults.

Previous studies have examined post-error behavioral adjustments from three perspectives (Danielmeier and Ullsperger, 2011). The first is post-error slowing (PES; Rabbitt, 1966), which denotes a delay in response during post-error trials compared with no-error trials. The second is post-error reduction of interference (PERI; Ridderinkhof, 2002), which pertains to the reduced effect of interference tasks, such as the Eriksen flanker task and Simon task, in post-error trials compared with no-error trials (Ridderinkhof, 2002; King et al., 2010). The third is post-error improvements in accuracy (PIA; Laming, 1968), which refers to increased accuracy of responses after error trials compared with no-error trials (Danielmeier et al., 2011).

Researchers have indicated a correlation between these post-error behavioral adjustments and two modes of cognitive control, namely, proactive and reactive control, within the dual mechanisms of control (DMC) framework (Braver et al., 2007; Ridderinkhof et al., 2011; Kirschner et al., 2021). Proactive control involves stable maintenance of task-related information and anticipatory cognitive processing, whereas, reactive control involves immediate attention to an object and instant cognitive processing (Braver, 2012). PES is closely correlated with reactive control because, under reactive control, the impact of an error directly influences individuals, which causes PES (King et al., 2010; Danielmeier and Ullsperger, 2011). In contrast, PERI and PIA are closely correlated with proactive control because, under proactive control, the “early selection” of task-related information is reconfirmed after an error, which suppresses the effect of interference and guides the correct response (Ridderinkhof, 2002; Ridderinkhof et al., 2002). However, Kirschner et al. (2021) described, thus far, only a few studies have examined the correlation between post-error behavioral adjustments and these two modes of cognitive control.

Research has reported developmental differences in the use of the two modes of cognitive control (Munakata et al., 2012). A transition occurs from the reactive control-dominant stage to the proactive control-dominant stage during the early stages of life (Chevalier, 2015; Gonthier et al., 2019). Conversely, the proactive control-dominant stage transitions to the reactive control-dominant stage in the later stages of life (Braver et al., 2005; Braver, 2012). Thus, reactive control is predominant in older adults, and may strongly influence post-error behavioral adjustment.

To the best of our knowledge, no previous study has examined post-error adjustment behaviors among older adults from the perspective of proactive and reactive control. To address this research gap, this study examined the correlation between post-error behavioral adjustments in older adults and these two modes of cognitive control. We developed an experiment in which young and older adult participants responded to an error when performing an experimental task, and compared the results between the two groups.

The experimental task was developed by modifying the error awareness task (EAT; Hester et al., 2005). The researchers instructed participants to push the right button with their preferred hand as quickly as possible when the Japanese character for the name of a color and the color of this stimulus were the same (go trial). Conversely, participants were asked to withhold their response when the Japanese character for the name of a color and the color of the stimulus were different (no-go trial). Response inhibition differed depending on the experimental condition. Consequently, the error responses differed depending on the imposed conditions. Moreover, participants were asked to press the left button using the same hand as soon as possible when an error occurred, under any condition. These tasks imitated the driving situation, where the right button corresponded to the accelerator and the left button corresponded to the brake pedal.

Additionally, we examined whether differences in error responses influenced post-error reactions. Ceccarini and Castiello (2018) reported response-dependent post-error changes in reaching movements, including grasping or not, indicating that the response time after an error is dependent on differences in error responses (e.g., motor components). In the case of a car driving situation, it is expected that the operation to switch to the brake pedal will be affected by the error responses when the accelerator is accidentally pressed down. For example, if the driver presses hard on the accelerator, there may be a delay in changing to the brake pedal.

In the present study, we focused on the difference in error responses as a difference in motor sequencing (i.e., the number of times a button was accidentally pushed). Changing the number of pushes is significant because pushing a button twice, for example, requires a longer motor sequencing process (*cf.* Fraser et al., 2010) and produces a heavier motor load than pushing it only once.

Previous studies investigating the motor load of cognitive tasks in older adults have used the motor-cognitive dual-task paradigm (Krampe, 2002; Woollacott and Shumway-Cook, 2002). Most of these studies examined gross motor skills such as walking, and only a few investigated fine motor skills, such as tapping a table (Albinet et al., 2006; Fraser et al., 2010; Pratt et al., 2014). In both gross and fine motor tasks, research has indicated a stronger effect of motor load on cognitive tasks in older adults compared with younger adults (Woollacott and Shumway-Cook, 2002; Kemper et al., 2003). These results suggest that differences in responses (i.e., motor sequencing) when making an error influence subsequent cognitive processing only in older adults.

To summarize, this study examined the characteristics of post-error adjustments in older adults from the viewpoint of cognitive control, using the go/no-go task to compare the response time after an error between young and older adults. By manipulating the differences in error responses (i.e., the number of times a button was accidentally pushed), we clarified the characteristics of post-error adjustments in older adults, in whom reactive control is likely to predominate.

This study presents the following hypotheses:

1. The response time after an error, compared with the response time for the go trial, will be longer in older than young adults because reactive control dominates the behavior of older adults, resulting in reduced readiness for errors. In contrast, proactive control is dominant in young adults, inducing a tendency to prepare for errors to minimize the impact of error responses.
2. The difference in error responses (i.e., the number of times a button was accidentally pushed) strongly influences older adults’ response time after an error because of reactive control dominance associated with the impact of immediately preceding error responses. It is expected that the motor load for pushing a button twice is heavier than that for pushing it once, which increases the post-error reaction time. This suggests that motor load during an error influences post-error behavioral adjustments in older adults.

## Materials and methods

### Participants

The study recruited 50 young adults (*M*_age_ = 21.6 years, age range = 18–25 years) and 50 older adults (*M*_age_ = 71.5 years, age range = 66–83 years). Each group had 25 men and 25 women. The participants reported to be healthy at the time of the study, although several of the older participants were reported having chronic illnesses. Throughout the instruction and practice trials of the experiment, it was confirmed that the participants had no significant visual or hearing impairments. In the experiment, the participants were instructed to perform the task using the preferred hand. As a result, all participants used their right hand.

All the older adults were pensioners living independently in local communities, registered with the Silver Human Resource Center (a day work agency for older adults in Japan), and typically engaged in light part-time work. All the young adults were university students. All participants received 1,000 yen per hour as a reward for participating in this study. The mean score on the Mini-Mental Status Examination (Folstein et al., 1975) for older adults was 29.1 (SD = 1.50), with a range of 27–30. We assumed that they had no cognitive impairment because their MMSE scores were 27 or higher and were living independently in the community. Their mean years of schooling was 13.3 years (SD = 2.5), and all of them had completed over 12 years of schooling. No statistically significant difference was observed in years of schooling between the young and older adults.

Informed consent was obtained from all participants prior to the study. The ethics committee of Ritsumeikan University approved this study.

### Equipment and procedure

**Figure 1:**
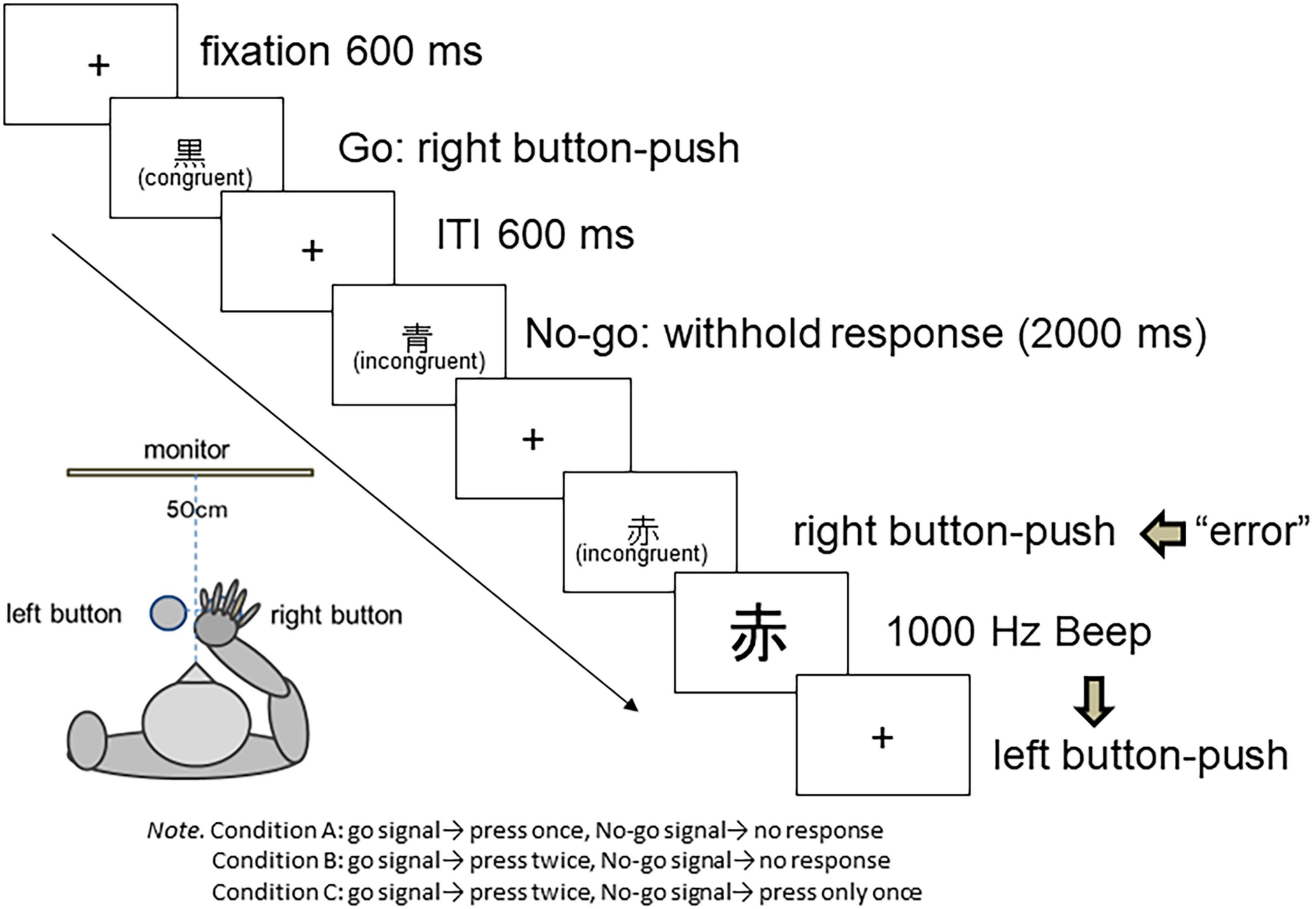
Graphical description of the experimental trials.

The task in this study was based on the EAT (Figure 1; Hester et al., 2005), which first presents a fixation point on the screen. The Japanese characters (Kanji) for red, blue, or black are presented in red, blue, or black, respectively, in a 100 points font (0.07 rad). If a character’s meaning and color were consistent (e.g., a black character reading “black”), it was regarded as a “go” stimulus and the participants pushed the right button in front of them. In contrast, if the character’s meaning and color were inconsistent (e.g., a blue character reading “black”), it was regarded as a “no-go” stimulus, and the participants were instructed to refrain from pushing the button for 2,000 ms (the response that should be suppressed differed depending on the experimental condition, as explained in section 2.3). The go-stimuli were presented in 25 of 30 trials, whereas the no-go stimuli were presented in the remaining trials. Participants were told to “immediately and accurately push the button.” If an error occurred, a beeping sound was presented at 1,000 Hz, and the font size of the visual stimulus was increased to 150 points (0.106 rad). The volume from the speakers reached approximately 70 dB at a distance of 50 cm. When an error occurred, participants were instructed to push the left button as quickly as possible, which stopped the beeping and started the subsequent regular trial. The interval between the trials was 600 ms.

The experiment comprised three conditions, each consisting of TEN successive blocks containing 30 trials with a 5-s interval between blocks. Eight practice trials were conducted for each condition. The participants were given a 3-min break after completing 300 trials.

Two round buttons (Johnan Corporation, Kyoto, Japan; operating pressure, 100 g; diameter, 10 cm) were used as response buttons. All stimuli were displayed on a monitor (I-O Data Device Inc., LCD-AD195GB, 19-inch model). Participants were seated approximately 50 cm away from the device; they used the palm of their right hand to push the response buttons located 5 cm apart. The experiment was controlled using a personal computer (Toshiba Corporation, Dynabook Satelite A50S).

### Experimental variables

We manipulated age (young or older adults) and three types of experimental conditions (A, B, and C), in which the number of button-pushes differed. We also examined whether the type of error response influenced post-error response time. We conducted the three conditions using a within-participants design in a counterbalanced order. The participants were instructed to push the left button as quickly as possible when an error occurred in any of the three conditions.

Condition A consisted of pushing the right button once when a go-stimulus was presented and refraining from pushing the right button for 2,000 ms when a no-go stimulus was presented.

Condition B consisted of pushing the right button twice when a go-stimulus was presented and refraining from pushing the right button for 2,000 ms when a no-go stimulus was presented. The motor load for the go-response under this condition was greater than that under condition A, because, compared to pushing the button once, pushing it twice entails longer motor sequencing and produces a heavier motor load.

Condition C consisted of pushing the right button twice when a go-stimulus was presented and pushing the right button only once when a no-go stimulus was presented.

Error responses were accidentally pushing the right button once when a no-go stimulus was presented in Conditions A and B, and accidentally pushing it twice when a no-go stimulus was presented in Condition C. The error response under Condition C imposed a heavier load on the respondents because they accidentally pushed the button twice, which included a longer motor sequencing, and produced a heavier motor load than pushing the button only once.

### Data analysis

We recorded the response times and number of error responses for each participant. The go response times were measured from the time the stimulus was presented to the first button press in all conditions. The number of error responses was taken as the error rate and the mean response time for each condition was calculated. Outliers defined as > 2 SD from the individual means were excluded. Statistical analyses were performed using R version 3.6.2. The alpha level of the statistical tests was set to 0.05.

We measured the time between committing the error and pushing the left button after the error. In condition C, committing the error was measured as the time when the second button was pressed. Based on that time, the response time after the error was measured. We expected general response times to be longer in older adults than young adults. We calculated the post-error reaction ratio as the difference between the mean response time for the go-stimulus and the mean reaction time when an error occurred using the formula below, by referring to the Stroop interference rate calculation method (Jensen, 1965). The Stroop interference rate was calculated by dividing the increase in response time under the interference condition by the control response time. In the current experiment, we calculated the post-error reaction ratio by dividing the increase in the response time after an error by the go-response time:


$$\mathrm{Post}-\mathrm{error reaction ratio}=\frac{{\bar{\mathrm{RT}}}_{\mathrm{post error}}-{\bar{\mathrm{RT}}}_{\mathrm{go}}}{{\bar{\mathrm{RT}}}_{\mathrm{go}}}$$

We hypothesized that the post-error reaction ratio for older adults would be higher than that for young adults. Moreover, the post-error reaction ratio of older adults under Condition C was expected to be significantly higher than that under Conditions A or B.

## Results

Table 1 presents the response times and error response rates for each condition. The response times for the go-stimuli were 566–586 ms and 677–700 ms in young and older adults, respectively; thus, older adults’ response times were generally longer (*F*(1, 98) = 46.02, *p* = 0.0000, *η*_G_^2^ = 0.28). The error response rate for the no-go stimuli was less than 3% under all conditions for both age groups. Therefore, we concluded that the experiment successfully replicated rare, sudden errors that occur in daily life for both age groups.

**Table 1:** Means and standard deviation of response times and error rates.

	**Go-response time (ms)**		**Error rate (%)**	
	**Young adults**	**Older adults**	**Young adults**	**Older adults**
Condition A	584 (91)	677 (76)	1.9 (1.7)	1.6 (1.1)
Condition B	587 (99)	700 (88)	2.0 (1.6)	1.4 (1.1)
Condition C	566 (96)	696 (96)	1.0 (1.0)	0.7 (0.7)

Go-response time: the interaction between age groups and conditions (*F*(2, 196) = 3.63, *p* = 0.0282, *η*_G_^2^ = 0.01) and the main effect of age group (*F*(1, 98) = 46.02, *p* = 0.0000, *η*_G_^2^ = 0.28) were significant. Error rate: the main effect of condition (*F*(2, 196) = 40.65, *p* = 0.0000, *η*_G_^2^ = 0.10) was significant (A > C, B > C). The range (min.–max.) of the number of times an error occurred for only those who made errors in all three conditions was as follows: For young adults, (1–16) in condition A, (1–16) in condition B, and (1–10) in condition C; and For older adults, (1–11) in condition A, (1–15) in condition B, and (1–6) in condition C.

**Figure 2:**
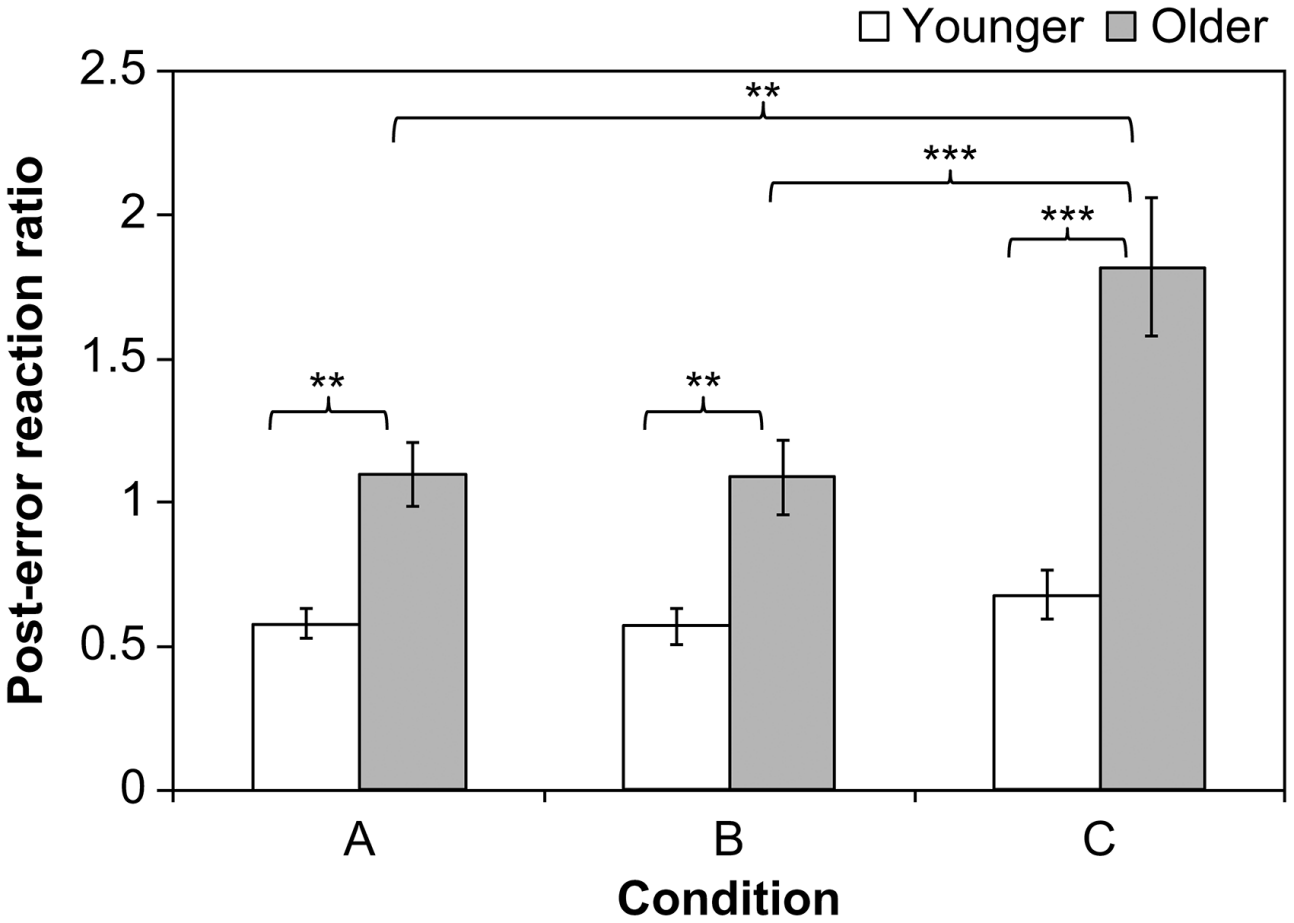
Mean post-error reaction ratio. Asterisks indicate significant differences (^**^p < 0.01, ^***^p < 0.001). Error bars indicate standard errors.

Figure 2 illustrates the differences in the post-error reaction ratios based on the three conditions. We conducted an analysis of variance for participants who committed errors under the three conditions (older adults: *N* = 28, young adults: *N* = 34). The results indicated that the main effects of age (*F*(1, 60) = 25.49, *p* = 0.0000, *η*_G_^2^ = 0.20) and condition (*F*(2, 120) = 13.22, *p* = 0.0000, *η*_G_^2^ = 0.08), and the interaction between age and condition (*F*(2, 120) = 8.64, *p* = 0.0003, *η*_G_^2^ = 0.057) were significant. The significant main effect of age suggests that the post-error reaction ratio for older adults was generally higher than that for young adults. We conducted a *t*-test using the Bonferroni method for multiple comparisons considering the significant differences found between the three conditions. The result indicated that the post-error reaction ratio under Condition C was higher than that under the other two conditions (between Conditions A and C: *t*(60) = 4.92, *p* = 0.0000; between Conditions B and C: *t*(60) = 3.96, *p* = 0.0002; between Conditions A and B: *t*(60) = 0.61, *p* = 0.5450).

The significant interaction suggests that the effects of the conditions differed between young and older adults. We analyzed the interactions and found no significant differences between the conditions in young adults (*F*(2, 66) = 0.82, *p* = 0.4445, *η*_G_^2^ = 0.01); however, a significant difference was observed among older adults (*F*(2, 54) = 4.63, *p* = 0.0001, *η*_G_^2^ = 0.15). The *t*-test indicated no significant differences between Conditions A and B. In contrast, the post-error reaction ratio under Condition C was higher than that under the other two conditions in older adults (between Conditions A and C: *t*(27) = 3.60, *p* = 0.0013; between Conditions B and C: *t*(27) = 4.67, *p* = 0.0002; between Conditions A and B: *t*(27) = 0.74, *p* = 0.4632). The results indicate that the post-error reaction ratio for older adults generally increased. Furthermore, the post-error reaction ratio in Condition C increased only for older adults.

## Discussion

This study examined the characteristics of post-error adjustment behaviors in older adults from the viewpoint of cognitive control, using the go/no-go task to compare the post-error reaction ratio between young and older adults. The results showed relatively larger post-error ratio to sudden errors among older adults compared with young adults. Furthermore, the difference in the error responses (i.e., accidentally pushing the right button once or twice) strongly influenced older adults’ post-error reaction ratio. These results suggested that the mode of reactive control may significantly influence post-error behavioral adjustments in older adults. These findings support the hypotheses, which were based on the DMC framework (Braver, 2012).

Research on the early stages of development can help elucidate aging-related changes. For example, children aged 6 years and older who have acquired proactive control are prepared for response switching in set-shifting tasks (Chevalier, 2015), which enables them to suppress the effects of interfering stimuli and prepare for events. Conversely, children aged less than 5 years are more strongly affected by such stimuli and events (Chevalier et al., 2015). Other studies have reported similar developmental changes in different tasks (Blackwell and Munakata, 2014; Chevalier et al., 2014; Voigt et al., 2014).

The results of the current study suggest a developmental change in the opposite direction, that is, a transition from the proactive to reactive control phase, as a result of aging. Older adults, who are in the reactive control-dominated stage, required more time to respond to sudden errors. Thus, proactive or reactive control (i.e., being prepared or unprepared for sudden errors) influenced the results.

The post-error reaction ratio for older adults under Condition C was higher than that under the other two conditions, which suggests that the difference in error responses (i.e., accidentally pushing the right button once or twice) highly influenced the post-error behavioral adjustments in older adults. Alternatively, the motor load for the go-response (Condition B) did not significantly influence post-error behavioral adjustments, which implies that the difference in motor load when making an error highly influenced post-error behavioral adjustments in older adults.

Previous studies have reported that differences in response type influence the results of cognitive tasks in older adults. For example, between two types of suppressions tasks, the Simon task (response suppression task) showed the effects of aging more strongly than the flanker task (interference suppression task; Kawai et al., 2012). Moreover, differences in response type were found to strongly influence response inhibition in older adults (Tsuchida et al., 2013; Tsuchida, 2017). These findings indicate that differences in response type (i.e., motor sequencing) when making an error influence subsequent cognitive processing in older adults.

The results of the present study also suggest that cognitive control problems may have an impact on daily life. Older adults who can adapt well to typical situations (go-stimulus settings) may be unable to adapt to sudden errors, which may result in serious accidents. For example, older adults often keep their feet on the accelerator even when they need to press the brake pedal (Hasegawa et al., 2020). Furthermore, the results suggest that in the older adults, differences in the way in which the accelerator pedal is pressed (e.g., motor factor) have a strong effect on the time it takes to press the brake pedal. However, these notions must be carefully tested in real-world settings, such as a driving simulator.

This study also has its limitations. First, the results of this study in terms of switching, which is one of the elements of the executive function (Miyake and Friedman, 2012) need to be examined. The target of this study was the switching of responses from the right button to the left button after an error. Although this study interpreted the results within the framework of post-error correction behaviors (e.g., Hasegawa et al., 2020), it may be necessary to examine the results within the framework of motor switching (e.g., Sombric and Torres-Oviedo, 2021). In any case, the results suggest that the older adults need more time to respond to unexpected events, such as sudden errors, and that differences in the motor level of error responses may strongly influence the motor switching time.

Second, the influence of the magnification of the visual stimulus and the beeping sound were presented at the same time during the error. The possibility that the delayed response to the left button is due to delayed responses to visual and auditory cues cannot be ruled out. It is necessary to examine the effects of visual and auditory cues separately from error responses. However, in the older adults, the differences in error responses (i.e., accidentally pushing the right button once or twice) had a strong influence on the reaction time to the left button. This suggests that the contents of error itself had an influence on the subsequent responses.

Third, there are issues related to error rates. The error rate in condition C was lower than in the other conditions. We cannot dismiss the possibility that this difference affected only the older adults. However, the error rate in all three conditions was less than 3% in this study. Since the error rates were generally low, we estimated it unlikely that this difference in error rates had a significant effect on the response time after an error. However, the possibility that the low error rates may cause the response times after errors to vary greatly from situation to situation cannot be excluded. It is necessary to reexamine this possibility by setting up an experiment in which the error rate is a little higher in the experimental setting. However, it is also true that we do not have the knowledge of how many errors would have allowed us to measure a stable post-error response time. Even in previous studies, the error rates seem to vary considerably from around 10 to 2% (e.g., Dudschig and Jentzsch, 2009; Jentzsch and Dudschig, 2009; Danielmeier and Ullsperger, 2011). It seems necessary to accumulate data on the present results as an example.

Finally, we discuss future research issues. One is the difference in strategies between younger and older adults in the go/no-go task. Comparing the reaction time and error rate in the go/no-go task between younger and older adults, we found that the older adults tended to make fewer errors even when their reaction time was slower. It is thought that the older adults responded carefully in order to avoid errors as much as possible in this kind of experimental task. The need to examine how such differences in strategies affect the way we respond to errors in daily life is indicated.

In conclusion, we found relatively longer reaction times to sudden errors among older adults compared with young adults. Furthermore, the difference in the error responses (i.e., accidentally pushing the right button once or twice) strongly influenced older adults’ response time after an error. Our results suggest that the shift from proactive to reactive control may significantly influence post-error behavioral adjustments in older adults.

## Data availability statement

The raw data supporting the conclusions of this article will be made available by the authors, without undue reservation.

## Ethics statement

The studies involving human participants were reviewed and approved by Ritsumeikan University. The patients/participants provided their written informed consent to participate in this study.

## Author contributions

NT conceived and planned the experiments and wrote the manuscript with input from all the authors. AK and MK performed the experiments and contributed to the interpretation of the results. NT and AK analyzed the data. All authors contributed to the article and approved the submitted version.

## Funding

This study was supported by Grants-in-Aid for Scientific Research in Japan (19K03272) and International Association of Traffic and Safety Sciences (1706C).

## Conflict of interest

The authors declare that the research was conducted in the absence of any commercial or financial relationships that could be construed as potential conflicts of interest.

## Publisher’s note

All claims expressed in this article are solely those of the authors and do not necessarily represent those of their affiliated organizations, or those of the publisher, the editors and the reviewers. Any product that may be evaluated in this article, or claim that may be made by its manufacturer, is not guaranteed or endorsed by the publisher.
